# Supplementary material for: Acceptance and compliance with micronutrient powder (MNP) among children aged 6–23 months in northern Nigeria
Source: PLOS Glob Public Health. 2022 Oct 17;2(10):e0000961. doi: 10.1371/journal.pgph.0000961 (PMC10022258; doi:10.1371/journal.pgph.0000961)
Supplement: S1 Dataset — (ZIP) [file pgph.0000961.s008.zip › S8_Dataset/Direct Observations.zip!/Direct Observations/180615_K_BK_SA_1_DO.pdf]

1.1.1 Instrument F –Direct Observation Form

**<<Instrument F Direct Observation Form>>**
**PI Name:** PragyaMathema,Stephen Kodish<br>**Study Title:** Formative research to develop an infant and young child feeding (IYCF) intervention with micronutrient powder (MNP) for children aged 6 – 23 months<br>**PI Version/Date:** Version1.0/January 24, 2015

Date: 18/06/15Location: Yamama Ketare

Data Collector: Samira Adama

Main goals of this direct observation are to answer the following questions:
1. In this household, what would be the best way(s) to give nutritional supplements to young children? Why?<br><br>2. In this household, describe the food sharing that you observe throughout the meal. Are there any predictable patterns?<br><br>3. In this household, who is favored and how is this favoritism expressed during meal preparation and eating?

Household Composition Table (Fill out as you do data collection)

INITIALS	SEX (M/F)	AGE (estimated)	Relationship to household	Description (not necessary)
SBB	M	14 months	Youngest child	Slim black boy without clothes
SBCY	F	16 years	Mother (2nd wife)	Slim and black with coffee brown and yellow wrapper
SOG	F	20 years	Step mother (1st wife)	Slim with orange and green wrapper
BGS	M	8years	Brother (2nd child)	Young boy with green shorts
BLGS	M	10years	1st child	Boy with light green shorts
BGN	F	17months	Second young daughter	Baby girl with no clothes
YGP	M	38years	Husband	Tall man wearing green and peach trousers
PAG	F	24years	Neighbor	Slim dark lady wearing ash and green attire with green scarf
SBGU	F	3years	4th child	Slim dark baby girl with unplaited hair

SCP	M	6years	3rd child	Slim dark boy with cream colored pants.

PARTICIPANT ID: __________

TIME	ACTOR INITIALS	RECIPIENT INITIALS	WHAT HAPPENED	FOOD	Reactivity
10:35	SBCY		Pounding grains	Millet	
10:40	SOG		Pounding grains	Millet	
10:45	SBB		Still pounding	Millet	
10:50	SBB		Eating biscuits	Biscuits	
10:55	SBB		Lying down on the mat		
11:00	SBB		Eating biscuits again	Biscuits	
11:05	SBB		Still eating biscuits	Biscuits	
11:10	SLGS		Carrying bucket with grains		
11:15	BGS		Picking biscuits from the SBB without permission	Biscuits	Quickly taking biscuits
11:20	SOG & PAG		Pounding millet again	Millet	
11:45	SBCY		Setting of fire		
11:50	SBCY		Carrying a bucket to fetch watch	water	Fetching of water
11:55	SBB		Started eating biscuits again		
11:55	SBCY		Carrying bucket of water		
11:55	SBCY		Washing of pot		

TIME	ACTOR INITIALS	RECIPIENT INITIALS	WHAT HAPPENED	FOOD	Reactivity
11:57	SBCY		Putting pot on fire with water		
12:00	TGP		Carrying cutlass to farm		
12:05	SBB		Eating biscuits		
12:05	SOG& PAG		Still pounding millet	Millet	
12:05	SBCY		Still pounding	Millet	
12:10	SBCY	SBB	Feeding the baby	Biscuit	
12:10	SBCY	SBB	Backing the baby		
12:15			Pot on the fire		
12:15	SBCY		Still pounding	Millet	
12:20	SBCY		Sieving millet with flour	Millet	
12:20	SBB		Crying		
12:20			Breastfeeding the child	Breast milk	
12:25	SBCY	SBB	Giving the baby water	water	
12:30			Continued breastfeeding	Breast milk	
12:30	SBCY	SBB	Backing the baby		
12:35	SBB		Backing the baby		
12:35	SBCY		Seiving millet and water, mixes it to form paste	Millet	
12:40	SBCY		Pounding the paste	Millet	
12:40	SBCY		Setting the fire again		

TIME	ACTOR INITIALS	RECIPIENT INITIALS	WHAT HAPPENED	FOOD	Reactivity
12:40	SBCY		Putting the pot on fire		
12:40	SBCY		Pounding the paste again	Millet	
12:40	SBB		Still lying on the mat		
12:40	SBB		Baby vomiting		
12:45			Molding millet into balls	Millet	
12:45	SBCY		Pounding millet with water	Millet	
12:45	SOG		Washing pots		
12:45	SBCY		Making millet into paste	Millet	
12:50	SBB		Playing with sticks		
12:50	SBCY		Pounding millet again	Millet	
12:50	SBCY		Molding millet into balls	Millet	
12:56	SBB		Still playing with sticks		
12:57	SBCY		Putting millet balls into pot		
1:00	SBB		Playing with eating bowl		
	SBCY		Pounding and mixing flour	Millet	
1:05	SBB		Still playing alone		
1:10	SBB		Crying while sitting		
	SBCY		Stirring the millet balls in the pot		
1:12			Preparing kunu for baby	Millet	
1:15	SBCY		Opening MNP for the baby with		Cutting and looking at the

TIME	ACTOR INITIALS	RECIPIENT INITIALS	WHAT HAPPENED	FOOD	Reactivity
			blade		data collector
	SBCY		Stirring the kunu to cool		Facing the data collector's direction
1:20	SBCY	SBB	Feeding the baby	Breast milk	
1:25	SBCY		Adding MNP in small portion of kunu	Millet	
1:27	SBCY	SBB	Feeding the baby kunu with MNP		Sitting close to me
1:29	SBCY	SBB	Forcing the child to finish it		
1:35	SBB		Crying and crying on the mat		
1:40	SBCY		Setting fire again		
1:40	SBB		Vomiting again		
1:45	SBCY	SBB	Giving the baby water		
1:50	SBCY	SBB	Backing the baby		
1:55	SBB		Lying on the mat		
2:00	SBB		Eating biscuits and lying	Biscuits	
2:05	BGN		Sharing biscuits with SBB	Biscuits	
2:10	SBCY	SBB	Feeding the child	Breast milk	
2:15	SBCY	SBB	Feeding the child	Breast milk	
2:20	SBCY		Removing millet balls from boiling water in the pot		

TIME	ACTOR INITIALS	RECIPIENT INITIALS	WHAT HAPPENED	FOOD	Reactivity
2:25	SBB		Crying while lying down		
2:25	SBCY		Pounding millet balls to make fura	Millet	
2:30	SBCY		Still Pounding millet balls to make fura	Millet	
2:30	SBB		Lying down on mat		
2:35	SBCY		Moulding the fura		
2:40	TGP		Carrying a bucket		
2:45	SBB		Crying on the mat		
2:50	SBCY	SBB	Backing the baby		
2:55	SOG &SBCY		Pounding sorghum	sorghum	
3:00	SBB		Eating kosai	Bean cake	
3:05	SBB		Eating kosai	Bean cake	
3:10	SBB		The baby is crying		
3:15	SBCY		Feeding the baby	Breast milk	
3:20	SBB		Lying on the mat		
3:25	SBB		The baby is crying		
3:30	SBCY	SBB	Feeding the baby	Breast milk	
3:35	SBCY	SBB	Backing the baby		
3:40	SBB		Taken along to fetch water		
3:45	SBB		Lying on the mat		
3:50	SBCY	SBB	Feeding the baby	Breast milk	

TIME	ACTOR INITIALS	RECIPIENT INITIALS	WHAT HAPPENED	FOOD	Reactivity
3:55	SBCY	SBB	Feeding the baby	Breast milk	
4:00	SBCY	SBB	Giving the baby water		
4:05	SBB		Baby is sleeping		
4:10	SBB		Baby is sleeping		
4:15	SBB		Baby is sleeping		
4:20	SBCY		Preparing fura with cow milk	Millet & cow milk	
4:25	SBGU	SBGU	Feeding her with fura		
4:30	SBB		Baby is sleeping		
4:35	SBCY		Preparing fura with milk		
4:40	SBB		Baby is sleeping		
4:40	SBCY		Washing plates		
4:43	SBCY		Adding water to furu		
4:48	SBB		Baby is still sleeping		
4:53	SBCY	SBB	Bathing the baby		
4:58	SBCY	SBB	Feeding the baby	fura	
5:03	SBCY	SBB	Still feeding the baby	fura	
5:12	SBCY	SBB	Rinsing the baby’s body		
5:17	SOG	BGS<br>SCP<br>SGBU<br>BGN	Sharing food among the children i.e SCP,SGBU AND BGN, separately	Sorghum	

5:22	SBCY	SBB	Feeding the baby	Breast milk	
5:27	SBB		Lying on SOG'S lap		
5:32	SBB	BGN	Playing with sister		
5:37	SBB	BGN	Playing with sister		
5:42	SBB	BGN	Eating Gwaben dawa from sister's plate	Gwaben dawa	
5:47	SBB	BGN	Feeding the baby	Breast milk	
5:52	SBCY	SBB	Feeding the baby	breast milk	
5:57	SBB		Lying on the bed		
6:02	SBB		Stooling on the bed		
6:07	SBB		Vomiting again		
6:12	SBCY	SBB	Backing the baby		
6:17	SBB		Crying on the cushon		
6:22	SBCY	SBB	Feeding the child	breast milk	
6:27	SBCY	SBB	Feeding the child	breast milk	
6:32	SBB		The child is sleeping		
6:37	SBB		The child is sleeping		
6:42	SBB		The child is sleeping		

6:52	SBB		The child is sleeping		
6:57	SBB		The child is sleeping		
7:02	SBB		The child is sleeping		
7:07	SBB		The child is sleeping		
7:12	SBB		The child is sleeping		
	SBCY		She is sharing the fura		
	SOG	BGN	Feeding her child		
7:19	SBB		Still sleeping on the bed		
7:22	SBB		Still sleeping on the bed		
	SBCY		Placing bucket and big storage bowls for storing water		
7:27	SBB		The child is still sleeping		
7:32	SBB		Started crying but still dozing off		
7:37	SBCY	SBB	Feeding the baby	Breast milk	
7:42	SBCY	SBB	Feeding the baby<br>Giving the child water	Breast milk	
7:42	SBB		Went back and started sleeping again		
7:47	SBB		The child is sleeping		
7:52	SBB		The child is still sleeping<br>No action from other household members because it was raining heavily		
7:57	SBB		The child is still sleeping		

8:02	SBB		The child is still sleeping		
8:07	SBB		The child is still sleeping		
8:12	SBB		The child is still sleeping		
8:17	SBB		The child is still sleeping		
8:22	SBB		The child is still sleeping		
8:27	SBB		The child is still sleeping		

Primary Questions (Complete after you complete data collection):

From what you have seen observing THIS HOUSEHOLD, please answer the following questions in detail:

Question	Write your detailed response here
1. In this household, what would be the best way(s) to give nutritional supplements such as an MNP to young children to ensure compliance?<br><br>Why?	The best and proficient way to give the MNP is through then mothers.<br><br>The child is so attached to the mother and she keeps curdling backing the baby. In essence, she would ensure proper administration and compliance.
2. In this household, who is favored during meal preparation or feeding?<br><br>How is this favoritism expressed?	The child is the most favored and the children are also favored then the parents.<br><br>The child is fed by the mother while she carries him on her laps and she ensures the baby feeds well to satisfaction before older children.
3. How could we promote the MNP supplement for this family that would attract their attention?	This MNP supplement for the family should be promoted.

Who would they listen to?	The father because he is always coming home and keeps an eye on the wives about the kids. The wives are generally answerable to their husbands and they obey all commands given to them by their husbands
4. In this household, what would be the best ways to promote the MNP but limit how much it would be shared?<br><br>Why?	Proper sensitization and creating awareness about the product to then mothers should be encouraged by the resource persons sharing although they do not have
5. What other issues and concerns did you see with this family that are important for us to consider with regard to introduction of an MNP?	Lack of food is a challenge to the family and also lack of proper sensitization about the product.

Why?	Because I observed, although being a special period (fasting), no attempt of cooking was made except the fura made and the children did not eat anything until the fura was prepared that was about 3:30pm
6. Based on your experience observing this child today, do you believe reactivity negatively impacted your ability to see the food-related behaviors of the child?<br><br>Please explain your answer in detail.	Not really, since the child was mostly fed by the mother and th other siblings fed on their own using separate bowls. But on the other hand, there was little food sharing between the child and the 2nd youngest child in the household.
